# Emerging Role of Immunotherapy in Neoadjuvant, Adjuvant, and Perioperative Management of Gastric Cancer

**DOI:** 10.7759/cureus.108998

**Published:** 2026-05-16

**Authors:** Praloy Basu

**Affiliations:** 1 Medical Oncology, Netaji Subhash Chandra Bose Cancer Hospital, Kolkata, IND

**Keywords:** gastric cancer, immune checkpoint inhibitors, immunotherapy, neoadjuvant treatment, perioperative therapy

## Abstract

Gastric cancer remains a major global cause of cancer mortality despite improvements in surgery, perioperative chemotherapy, radiotherapy, and supportive care. For resectable locally advanced gastric and gastroesophageal junction adenocarcinoma, perioperative chemotherapy with fluorouracil, leucovorin, oxaliplatin, and docetaxel (FLOT) has become a major standard of care; however, recurrence remains frequent, reflecting the persistence of micrometastatic disease and biological resistance. Immune checkpoint inhibitors have transformed the management of advanced gastric cancer and are now being investigated in curative-intent settings. The rationale for earlier use is strong: intact tumor antigen, preserved lymphatic priming, and potential eradication of micrometastases may enhance immune-mediated control. This review discusses the emerging role of immunotherapy in neoadjuvant, adjuvant, and perioperative gastric cancer management, with a particular focus on pivotal trials including DANTE, Neoadjuvant Nivolumab Plus Ipilimumab and Adjuvant Nivolumab (NEONIPIGA), KEYNOTE-585, ATTRACTION-5, CheckMate-577, and MATTERHORN. MATTERHORN represents the most important recent phase III advance, showing that perioperative durvalumab plus FLOT significantly improves event-free survival compared with FLOT alone in resectable gastric or gastroesophageal junction adenocarcinoma. Future integration of immunotherapy will require careful biomarker selection, interpretation of survival endpoints, toxicity management, and adaptation to regional treatment standards.

## Introduction and background

Gastric cancer remains one of the most important malignancies worldwide, both because of its frequency and because of its persistently high mortality. Globally, it ranks among the most commonly diagnosed cancers and remains a leading cause of cancer-related death [1]. Although incidence has declined in some regions because of improved food preservation, reduced *Helicobacter pylori* prevalence, and better public health measures, the global burden remains substantial, particularly in East Asia, Eastern Europe, and parts of South America. In many patients, diagnosis occurs at a locally advanced or metastatic stage, and even among those undergoing curative-intent resection, relapse remains common.

The management of resectable gastric cancer has changed considerably over the past two decades. Surgery alone is inadequate for most patients with locally advanced disease because both locoregional and distant relapses occur frequently. Western practice was strongly influenced by the MAGIC trial, which established perioperative chemotherapy as superior to surgery alone in resectable gastric, gastroesophageal junction, and lower esophageal adenocarcinoma [2]. Subsequently, the FLOT4-Arbeitsgemeinschaft Internistische Onkologie (AIO) trial demonstrated that perioperative fluorouracil, leucovorin, oxaliplatin, and docetaxel (FLOT) improved survival compared with older epirubicin-based regimens, thereby establishing FLOT as a contemporary perioperative standard in fit patients [3].

Adjuvant approaches have also contributed to improved outcomes. In East Asia, where D2 lymphadenectomy is routinely performed, trials such as ACTS-GC and CLASSIC established adjuvant fluoropyrimidine-based chemotherapy as beneficial after curative gastrectomy [4,5]. In North America, postoperative chemoradiotherapy gained acceptance after INT-0116, although its role has become more selective in the era of adequate D2 surgery and effective systemic therapy [6]. Despite these advances, a large proportion of patients relapse, commonly with distant metastases, indicating that conventional chemotherapy does not adequately eradicate micrometastatic disease.

The success of immune checkpoint inhibitors in advanced gastric cancer has provided a strong rationale for their movement into earlier disease stages. In metastatic disease, nivolumab plus chemotherapy improved survival in CheckMate-649, and pembrolizumab plus chemotherapy improved outcomes in KEYNOTE-859 [7,8]. These trials confirmed that immune checkpoint blockade has clinically meaningful activity in gastric and gastroesophageal junction adenocarcinoma, especially in biomarker-enriched populations. The key question is whether introducing immunotherapy earlier, before or after surgery, can increase cure rates rather than merely prolong survival in advanced disease.

Biologic rationale for early immunotherapy in gastric cancer

The biological rationale for immunotherapy in gastric cancer is supported by molecular heterogeneity and variable immune responsiveness. The Cancer Genome Atlas classified gastric adenocarcinoma into molecular subtypes, including Epstein-Barr virus (EBV)-positive tumors, microsatellite instability-high tumors, genomically stable tumors, and chromosomal instability tumors [9]. Among these, EBV-positive and high microsatellite instability (MSI-H) tumors are particularly relevant to immunotherapy. EBV-positive tumors often show prominent immune cell infiltration and PD-L1/PD-L2 amplification, while MSI-H tumors have high tumor mutational burden and abundant neoantigens. These biological features create a permissive environment for immune checkpoint blockade.

Further molecular analyses have reinforced the clinical relevance of these subtypes. MSI-H gastric cancers show greater sensitivity to immune checkpoint inhibitors, while EBV-positive tumors also appear immunologically active [10]. Across tumor types, deficient mismatch repair and MSI-H status predict response to PD-1 blockade, supporting tissue-agnostic use of immunotherapy in this molecular context [11]. Tumor mutational burden, immune gene signatures, and T-cell-inflamed phenotypes have also been associated with response, although none is sufficiently reliable alone in perioperative gastric cancer [12,13].

The neoadjuvant setting may be particularly suitable for immunotherapy. When the primary tumor is still present, it provides a large reservoir of tumor antigen. Antigen-presenting cells can process tumor-derived antigens and activate tumor-specific T cells within intact lymphatic networks. This may generate systemic immunity capable of targeting micrometastatic disease. Preclinical work has shown that neoadjuvant checkpoint blockade can generate stronger systemic antitumor immune responses than adjuvant-only treatment [14]. This is relevant in gastric cancer, where distant relapse is common even after apparently complete resection.

Chemotherapy may also enhance immunotherapy. Cytotoxic agents can induce immunogenic cell death, increase antigen release, reduce immunosuppressive cellular populations, and alter the tumor microenvironment [15]. Oxaliplatin, in particular, has been associated with immunogenic cell death, while taxanes may modulate antigen presentation and immune infiltration. Thus, the combination of FLOT and immune checkpoint blockade has a plausible biological basis: chemotherapy may expose tumor antigens and remodel the microenvironment, while PD-1 or PD-L1 blockade may prevent exhaustion of activated antitumor T cells.

Current standards before immunotherapy integration

Before discussing immunotherapy trials, it is important to contextualize them against existing standards. For fit patients with resectable locally advanced gastric or gastroesophageal junction adenocarcinoma in many Western settings, perioperative FLOT is the preferred standard. The FLOT4-AIO trial demonstrated improved pathological response, R0 resection, progression-free survival, and overall survival compared with epirubicin-based therapy [3]. However, FLOT is intensive and may not be suitable for frail patients, elderly patients with comorbidities, or those with poor nutritional status.

In East Asian practice, upfront surgery followed by adjuvant chemotherapy remains common, particularly after high-quality D2 gastrectomy. The Adjuvant Chemotherapy Trial of TS-1 for Gastric Cancer (ACTS-GC) demonstrated improved survival with adjuvant S-1 after D2 gastrectomy [4], while CLASSIC demonstrated benefit with adjuvant capecitabine plus oxaliplatin [5]. These regional differences matter because immunotherapy trials have used different backbones, different surgical standards, and different patient populations. A practice-changing regimen within a perioperative FLOT-based framework may not automatically replace adjuvant strategies in regions where surgery-first treatment is standard.

## Review

Neoadjuvant immunotherapy: concept and early evidence

The neoadjuvant use of immunotherapy has several potential goals: increasing tumor regression, improving R0 resection rates, eradicating micrometastatic disease early, and generating durable immune memory. Pathological complete response is an attractive endpoint because it provides early evidence of tumor eradication, but gastric cancer differs from breast or rectal cancer in that pathological complete response (pCR) has not yet been validated as a universal surrogate for overall survival. This distinction is important when interpreting early-phase studies.

The DANTE trial is one of the most informative perioperative immunotherapy studies before MATTERHORN. DANTE evaluated atezolizumab added to perioperative FLOT in resectable esophagogastric adenocarcinoma. Interim results showed that atezolizumab plus FLOT was feasible and improved pathological regression compared with FLOT alone [16]. The trial suggested that PD-L1 blockade could deepen tumor response when combined with a modern chemotherapy backbone. However, DANTE was not initially powered to definitively establish a survival benefit in its phase II component, and longer follow-up is required.

A different and highly important approach is biomarker-selected neoadjuvant immunotherapy. The Neoadjuvant Nivolumab Plus Ipilimumab and Adjuvant Nivolumab (NEONIPIGA) phase II study evaluated nivolumab plus ipilimumab followed by surgery and adjuvant nivolumab in localized deficient mismatch repair (dMMR) and MSI-H gastric or gastroesophageal junction adenocarcinoma [17]. The results were striking, with very high pathological complete response rates. This trial is clinically important because dMMR/MSI-H tumors may derive limited benefit from perioperative chemotherapy but substantial benefit from immune checkpoint blockade. NEONIPIGA therefore supports the idea that some patients may eventually be treated with chemotherapy-free neoadjuvant immunotherapy, although this approach requires careful validation and multidisciplinary oversight.

The implications of NEONIPIGA extend beyond MSI-H disease. It demonstrates that molecular stratification is essential in perioperative gastric cancer. Treating all gastric cancers as a single entity risks overtreating some patients and undertreating others. Future neoadjuvant strategies are likely to divide patients into biomarker-defined groups: MSI-H/dMMR tumors may be candidates for immunotherapy-dominant approaches, HER2-positive tumors for HER2-directed chemoimmunotherapy, and biomarker-unselected tumors for chemoimmunotherapy combinations.

KEYNOTE-585: lessons from a negative survival trial

KEYNOTE-585 was one of the first major phase III trials to test perioperative immunotherapy in resectable gastric and gastroesophageal junction adenocarcinoma. It evaluated pembrolizumab plus chemotherapy versus placebo plus chemotherapy in the neoadjuvant and adjuvant setting [18]. The trial demonstrated a clear improvement in pathological complete response with Pembrolizumab. However, event-free survival and overall survival did not meet the prespecified thresholds for statistical significance at the reported analysis.

KEYNOTE-585 is important because it cautions against assuming that improved pCR automatically translates into improved survival. Several explanations are possible. First, the chemotherapy backbone in the global population included cisplatin-based regimens, which may be less effective than FLOT. Second, gastric cancer is heterogeneous, and the benefit may have been diluted in biomarker-unselected patients. Third, the postoperative component of perioperative therapy is often difficult to deliver after gastrectomy because of postoperative recovery, nutritional compromise, and treatment-related toxicity. Fourth, subsequent therapies after recurrence may influence overall survival.

The trial also raises questions about endpoint selection. Event-free survival may be affected by progression before surgery, inability to undergo surgery, recurrence after surgery, and death. In perioperative trials, these components are clinically meaningful but heterogeneous. A therapy that increases pCR may still fail to improve event-free survival (EFS) if postoperative relapse patterns are unchanged or if treatment delays offset early benefit. Therefore, KEYNOTE-585 remains a critical reference point: it confirms biological activity but does not establish pembrolizumab-based perioperative therapy as a universal standard.

MATTERHORN: design, results, and clinical meaning

MATTERHORN is currently the most important perioperative immunotherapy trial in resectable gastric and gastroesophageal junction adenocarcinoma. It evaluated durvalumab, an anti-PD-L1 antibody, in combination with perioperative FLOT chemotherapy compared with placebo plus FLOT [19,20]. The trial is particularly important because it used FLOT, the most widely accepted perioperative chemotherapy standard for fit patients in many regions.

MATTERHORN enrolled patients with resectable gastric or gastroesophageal junction adenocarcinoma and randomized them to receive durvalumab plus FLOT or placebo plus FLOT in the perioperative setting. The treatment strategy included preoperative therapy, surgery, postoperative chemotherapy, and continued immunotherapy/placebo according to protocol. The primary endpoint was event-free survival, with key secondary endpoints including pathological complete response, overall survival, R0 resection, and safety.

The published phase III results showed that perioperative durvalumab plus FLOT significantly improved event-free survival compared with FLOT alone [20]. This distinguishes MATTERHORN from KEYNOTE-585 and makes it the first major global phase III trial to demonstrate a statistically significant EFS advantage for immunotherapy added to perioperative chemotherapy in resectable gastric or gastroesophageal junction cancer. The improvement in EFS is clinically meaningful because it reflects fewer events such as progression, recurrence, or death during the perioperative disease course.

MATTERHORN also demonstrated improved pathological response. The addition of durvalumab increased pathological complete response compared with FLOT alone. This finding is important not only as a marker of local tumor eradication but also as evidence that PD-L1 blockade can enhance the cytotoxic and immune-mediated effects of FLOT. Major pathological response and tumor regression were also improved, supporting the biological activity of the regimen.

The surgical findings are equally important. One of the major concerns with neoadjuvant immunotherapy is that immune-related inflammation, fibrosis, or toxicity might delay surgery or increase postoperative complications. MATTERHORN did not show a prohibitive surgical safety signal. Resection remained feasible, and the addition of durvalumab did not appear to compromise curative surgery. This is crucial because any perioperative systemic strategy must improve oncologic outcomes without reducing the chance of safe, complete resection.

The safety profile was consistent with expectations for FLOT and durvalumab. FLOT is associated with hematologic toxicity, neuropathy, gastrointestinal toxicity, fatigue, and infection risk. Durvalumab adds the possibility of immune-related adverse events, including thyroiditis, pneumonitis, hepatitis, colitis, adrenal insufficiency, and dermatologic toxicity. In MATTERHORN, toxicity was manageable in the context of a clinical trial. However, translation into routine practice will require careful patient selection, early recognition of immune-related adverse events, and close coordination between medical oncologists, surgeons, gastroenterologists, endocrinologists, and perioperative care teams.

The success of MATTERHORN may reflect several factors. The first is the FLOT backbone. FLOT is more active than older perioperative regimens and may provide stronger tumor antigen release and immune priming. The second is the use of PD-L1 blockade, which may preserve PD-L2-PD-1 interactions differently from PD-1 inhibitors, although the clinical relevance of this distinction remains uncertain. The third is trial design: MATTERHORN used a contemporary perioperative framework and included event-free survival as a primary endpoint, allowing it to capture clinically relevant benefits across the treatment continuum.

The comparison between MATTERHORN and KEYNOTE-585 will shape future clinical discussion. KEYNOTE-585 improved pCR without clearly improving survival, while MATTERHORN improved EFS. This suggests that not all chemoimmunotherapy regimens are equivalent. The chemotherapy partner, patient selection, immunotherapy agent, duration of therapy, and trial population all matter. It also emphasizes that pCR alone should not be used as the sole basis for changing practice in gastric cancer.

In clinical terms, MATTERHORN may redefine the perioperative standard for fit patients with resectable gastric or gastroesophageal junction adenocarcinoma who are candidates for FLOT. However, several questions remain. The magnitude of benefit across PD-L1 subgroups, MSI-H tumors, EBV-positive tumors, geographic regions, Lauren histology, nodal status, and tumor location requires careful interpretation. Whether all patients need postoperative immunotherapy, whether ctDNA can guide escalation or de-escalation, and whether MSI-H patients require chemotherapy at all remain unresolved. Nonetheless, MATTERHORN provides the strongest phase III evidence to date supporting perioperative chemoimmunotherapy in this disease.

Adjuvant immunotherapy

The adjuvant setting differs biologically from the neoadjuvant setting because the gross tumor has already been removed. The goal is the eradication of microscopic residual disease. In principle, immunotherapy may be effective in this setting, but the absence of the primary tumor may reduce antigen availability, and postoperative immune suppression or nutritional compromise may affect treatment delivery.

CheckMate-577 established adjuvant nivolumab as a standard in patients with resected esophageal or gastroesophageal junction cancer who had residual pathological disease after neoadjuvant chemoradiotherapy [21]. Although this trial was not designed for gastric cancer specifically, it is highly relevant to gastroesophageal junction adenocarcinoma and supports the broader concept that immune checkpoint blockade can improve outcomes in minimal residual disease.

In contrast, ATTRACTION-5 tested adjuvant nivolumab plus chemotherapy after D2 or more extensive lymph-node dissection in pathological stage III gastric or gastroesophageal junction cancer and did not show a significant benefit [22]. This negative result highlights the complexity of adjuvant immunotherapy in gastric cancer. Possible explanations include differences in tumor biology, postoperative immune environment, chemotherapy backbone, geographic population, and biomarker distribution. ATTRACTION-5 also suggests that simply adding immunotherapy to adjuvant chemotherapy after surgery may be less effective than giving immunotherapy while the primary tumor is still present.

These divergent findings support a central hypothesis: immunotherapy may be more effective when administered before surgery or as part of a perioperative strategy rather than only after resection. The intact tumor may be necessary for optimal immune priming, and neoadjuvant therapy may allow earlier treatment of micrometastatic disease.

Biomarkers and patient selection

Biomarker selection is essential if immunotherapy is to be used rationally in curative-intent gastric cancer. PD-L1 combined positive score is widely used in advanced disease, but its role in perioperative therapy remains less certain. PD-L1 expression is heterogeneous, may vary between primary and metastatic sites, and may be influenced by chemotherapy and inflammation. Assay differences also complicate interpretation [23].

MSI-H/dMMR status is the most clinically actionable biomarker. MSI-H tumors are highly sensitive to immune checkpoint blockade and may be relatively less responsive to conventional perioperative chemotherapy. Trials such as NEONIPIGA suggest that immunotherapy-based approaches can achieve major pathological responses in this subgroup [17]. In the future, MSI-H status should be assessed before treatment planning in resectable disease, not only after surgery.

Tumor mutational burden is another potential biomarker, but its role in gastric cancer remains less standardized [24]. Immune gene expression profiles, T-cell receptor clonality, EBV status, and tumor-infiltrating lymphocyte patterns may further refine selection. Circulating tumor DNA is particularly promising for perioperative decision-making. Postoperative ctDNA positivity may identify patients at high risk of relapse who could benefit from intensified adjuvant therapy, whereas ctDNA clearance after neoadjuvant treatment may help identify excellent responders [25].

Combination strategies beyond PD-1/PD-L1 blockade

Future perioperative therapy will likely involve rational combinations. HER2-positive gastric cancer is one major area. In metastatic HER2-positive disease, trastuzumab plus chemotherapy combined with pembrolizumab has shown high response rates, supporting immune synergy between HER2-directed therapy and checkpoint blockade [26]. Whether this strategy can improve cure rates in resectable HER2-positive gastric cancer is an important research question.

Radiotherapy may also enhance immunotherapy through antigen release, interferon signaling, and modulation of the tumor microenvironment [27]. This may be particularly relevant for gastroesophageal junction tumors, where chemoradiotherapy is commonly used in some regions. However, the optimal integration of radiation with immunotherapy in gastric cancer remains undefined.

Novel strategies include dual checkpoint blockade, LAG-3 or TIGIT inhibition, cancer vaccines, adoptive cellular therapy, and microbiome modulation. These approaches remain investigational but may become relevant for biomarker-defined or immunotherapy-resistant subgroups.

Toxicity, surgical coordination, and practical issues

Perioperative immunotherapy requires careful toxicity management. Immune-related adverse events may affect endocrine organs, liver, lungs, skin, gastrointestinal tract, kidneys, and nervous system [28]. In the perioperative setting, these toxicities can complicate surgery, anesthesia, nutrition, and postoperative recovery. For example, immune-mediated colitis may worsen nutritional status, hepatitis may delay chemotherapy, pneumonitis may increase anesthetic risk, and adrenal insufficiency may present as perioperative hypotension.

Therefore, multidisciplinary coordination is essential. Patients should be assessed before each cycle for symptoms of immune toxicity. Surgeons must be aware of immunotherapy exposure, and anesthetic teams should consider endocrine complications such as adrenal insufficiency or thyroid dysfunction. Postoperative recovery should be closely monitored, especially because gastrectomy itself is associated with nutritional compromise, weight loss, and reduced tolerance of adjuvant therapy.

Cost and access are also major issues, especially in low- and middle-income settings. FLOT itself requires good supportive care, and adding immunotherapy increases financial toxicity. Biomarker-directed use may help optimize value by identifying patients most likely to benefit.

Future directions

The future of immunotherapy in resectable gastric cancer will likely be individualized. For biomarker-unselected, fit patients, perioperative durvalumab plus FLOT may become a major standard if regulatory and guideline bodies adopt MATTERHORN results. For MSI-H/dMMR tumors, chemotherapy-free or chemotherapy-minimized immunotherapy strategies deserve further evaluation. For HER2-positive disease, HER2-directed chemoimmunotherapy may become a distinct pathway. For frail patients, less intensive regimens will be needed.

ctDNA-guided treatment is likely to become increasingly important. A patient who remains ctDNA-positive after surgery despite perioperative therapy may require escalation, whereas a patient with pCR and ctDNA clearance may be a candidate for de-escalation. Adaptive trials incorporating molecular response, pathological response, and ctDNA dynamics may help avoid both overtreatment and undertreatment.

## Conclusions

Immunotherapy is reshaping the curative-intent management of gastric and gastroesophageal junction adenocarcinoma. Early trials established biological activity, but KEYNOTE-585 showed that improved pathological response alone is insufficient to define a new standard. MATTERHORN represents a major step forward because perioperative durvalumab plus FLOT improved event-free survival compared with FLOT alone while maintaining surgical feasibility. This positions perioperative chemoimmunotherapy as a likely new direction for fit patients with resectable disease.

However, several issues remain unresolved, including optimal biomarker selection, the role of immunotherapy in MSI-H disease, the value of postoperative treatment, ctDNA-guided escalation, and applicability across regions with different surgical and systemic therapy standards. The next phase of progress will depend on integrating immunotherapy with precision oncology rather than applying it uniformly to all patients.
